# Improving Presentation Attack Detection Classification Accuracy: Novel Approaches Incorporating Facial Expressions, Backdrops, and Data Augmentation

**DOI:** 10.3390/s25072166

**Published:** 2025-03-28

**Authors:** Tayyaba Riaz, Adeel Anjum, Madiha Haider Syed, Semeen Rehman

**Affiliations:** 1 Institute of Information Technology, Quaid-e-Azam University Islamabad, Islamabad 45320, Pakistan; tayyabariaz@iit.qau.edu.pk (T.R.); aanjum@qau.edu.pk (A.A.); 2 Institute of Computer Technology, Technical University of Vienna (TU Wien), 1040 Vienna, Austria

**Keywords:** sparse learning, data augmentation, one-class domain adaptation, adversarial training, knowledge distillation, decision-making accuracy, face presentation attack detection

## Abstract

In the evolving landscape of biometric authentication, the integrity of face recognition systems against sophisticated presentation attacks (PAD) is paramount. This study set out to elevate the detection capabilities of PAD systems by ingeniously integrating a teacher–student learning framework with cutting-edge PAD methodologies. Our approach is anchored in the realization that conventional PAD models, while effective to a degree, falter in the face of novel, unseen attack vectors and complex variations. As a solution, we suggest a novel architecture where a teacher network, trained on a comprehensive dataset embodying a broad spectrum of attacks and genuine instances, distills knowledge to a student network. The student network, specifically focusing on the nuanced detection of genuine samples in target domains, leverages minimalist yet representative attack data. This methodology is enriched by incorporating facial expressions, dynamic backgrounds, and adversarially generated attack simulations, aiming to mimic the sophisticated techniques attackers might employ. Through rigorous experimentation and validation on benchmark datasets, our results manifested a substantial leap in classification accuracy, particularly for those samples that have traditionally posed a challenge. The newly proposed model, which can not only effectively outperform existing PAD solutions, but also achieve admirable flexibility and applicability to novel attack scenarios, truly demonstrates the power of the proposed teacher–student framework. This paves the way for improved security and trustworthiness in the area of face recognition systems and the deployment of biometric technologies.

## 1. Introduction

In today’s digital landscape, the seamless integration of technology into everyday life has heightened the demand for secure and reliable authentication mechanisms [1]. Among various biometric authentication techniques [2,3], face recognition technology stands out as an efficient and user-friendly solution, bridging the gap between physical and digital identities. However, face recognition systems are increasingly vulnerable to sophisticated presentation attacks (PAD) [4], where adversaries exploit advanced attack presentation (AP) techniques such as high-resolution video replays, 3D masks, and AI-generated synthetic images to bypass authentication mechanisms [5]. These threats are continuously evolving, demanding robust, adaptive, and future-proof PAD strategies capable of mitigating both known and emerging attack vectors. The dynamic nature of such attacks underscores the pressing need for more advanced countermeasures that not only address current vulnerabilities, but also anticipate future threats.

A major challenge in enhancing PAD systems lies in their ability to effectively detect and neutralize sophisticated presentation attacks [6]. Traditional PAD approaches, though effective to a certain extent, primarily rely on data-driven models trained on specific attack patterns, limiting their ability to generalize beyond known threats. As a result, these systems struggle to counter novel attack presentation (AP) techniques [7], making them a significant security vulnerability. The inability to adapt to new forms of attacks poses a serious risk to critical security infrastructures, financial systems, and sensitive data repositories. Therefore, ensuring the resilience and robustness of face recognition technologies is no longer just a technical challenge, but a crucial security imperative. As biometric authentication becomes increasingly embedded in global security frameworks [8], maintaining its integrity against ever-evolving cyber threats is paramount [9]. The motivation behind this research stems from this urgency, driving the need for more intelligent, adaptive, and scalable PAD solutions.

Recent studies have explored similar approaches to enhance PAD systems. For instance, ref.  [10] proposed a one-class knowledge distillation framework for face PAD, where a teacher network is trained on source domain data, and a student network learns to mimic the teacher’s representations only using genuine face samples from the target domain. During testing, the similarity between the teacher and student network representations is used to distinguish between genuine and attack samples. This method showed improved performance in cross-domain PAD scenarios. Another notable work by [11] introduced a meta-teacher framework for face anti-attack presentation (AP), where a meta-teacher is trained to supervise PA detectors more effectively. The meta-teacher learns to provide a better-suited supervision than handcrafted labels, significantly improving the performance of PA detectors. Building upon these advancements, our research integrates the teacher–student learning paradigm with PAD methodologies to enhance the adaptability and robustness of PAD systems against evolving presentation attacks. This integration aims to improve the generalization capabilities of PAD systems, ensuring their effectiveness in diverse and unseen attack scenarios. Another notable study [12] introduced a dual-teacher knowledge distillation method, incorporating perceptual and generative knowledge to improve generalization across different attack types, while leveraging domain alignment techniques to mitigate training instability. Furthermore, the learning meta-patterns [13] approach enhances the robustness of PAD systems by focusing on intrinsic attack presentation (AP) cues rather than superficial characteristics, thus improving the generalization capabilities. Additionally, federated learning [14] has been explored as a means to strengthen face PAD models while preserving data privacy, allowing multiple entities to collaboratively train detection systems without sharing raw data.

**Figure 1:**
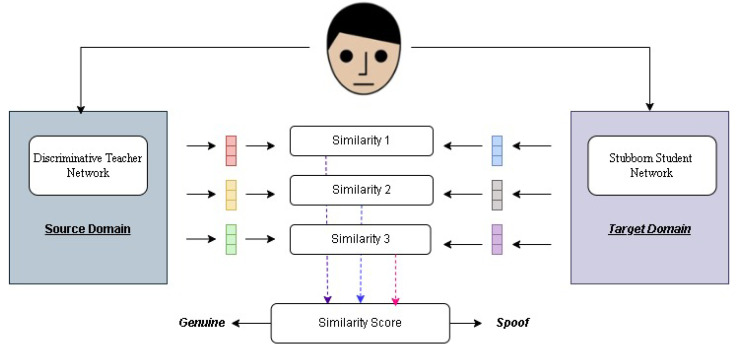
We train source and target domains, using the similarity of their extracted features as the inference score during testing.

These advancements have collectively contributed to the ongoing evolution of PAD technologies, reinforcing the necessity for adaptive and intelligent defense mechanisms. Our study makes a significant contribution to biometric security by proposing an innovative integration of a teacher–student learning framework with state-of-the-art PAD methodologies. The proposed approach aims to enhance the adaptability and accuracy of PAD systems, enabling them to effectively counter both known and emerging attack presentation (AP) attacks. By leveraging the deep knowledge of a teacher network—trained on an extensive dataset of both genuine and synthetic attack samples—the model guides a student network to specialize in recognizing subtle and complex facial features within targeted domains (refer to Figure 1). This strategic fusion bridges the gap between traditional PAD models and adaptive learning mechanisms, ensuring that the system remains resilient against evolving threats. Additionally, this framework lays the foundation for a scalable and adaptable security blueprint, enabling future research to build on these advancements, for more robust and trustworthy biometric authentication systems.

The rapid evolution of attack presentation (AP) techniques [15], combined with the widespread adoption of face recognition systems in critical sectors, necessitates a shift from reactive security measures to proactive, learning-driven approaches. Traditional static defense mechanisms are no longer sufficient; modern security demands intelligent, self-improving systems that continuously adapt to new attack methodologies. Ensuring the integrity and trustworthiness of biometric authentication systems [16] is essential for securing applications ranging from personal devices to global security infrastructures. Addressing this challenge requires moving beyond conventional PAD methods and implementing advanced machine-learning-driven solutions capable of predicting, evolving with, and mitigating emerging threats. This research directly addresses these concerns by leveraging cutting-edge AI-driven PAD methodologies, offering a scalable, resilient, and adaptable approach to biometric security.

The contributions of our research to the biometric security field and face presentation attack detection systems, which are different from the present literature, can be summarized in the following bullet points:

- Innovative Integration of Teacher–Student Learning Framework: We introduce a new application of the teacher–student learning paradigm to PAD systems, using the extensive knowledge of a teacher network trained on various real and synthetic attack vectors to guide a student network in accurately identifying genuine facial features.
- Enhanced Adaptability to Novel Attack Presentation (AP) Attacks: Our methodology significantly enhances the adaptability of PAD systems, enabling them to effectively counter both known and emergent attack presentation (AP) attacks. This adaptability is crucial for maintaining the integrity of biometric authentication systems amid a constantly changing environment of threats.
- Scalable and Adaptable Blueprint for Future Research: The proposed integration of machine learning frameworks with advanced PAD techniques provides a scalable and adaptable blueprint for future endeavors in biometric security. This contribution lays the groundwork for further innovations in the domain, potentially leading to more resilient and trustworthy authentication systems.
- Empirical Validation Against State-of-the-Art Solutions: Through rigorous experimentation and validation on benchmark datasets, our research demonstrated superior performance and efficacy in detecting attack presentation (AP) attacks, as compared to previous solutions. This empirical evidence substantiates the practical applicability and effectiveness of our proposed methodology.
- Fresh Perspective on Biometric Authentication Vulnerabilities: By synergizing advanced PAD methodologies with a teacher–student learning framework, our study offers a fresh perspective on addressing the vulnerabilities inherent in biometric authentication systems. This contribution provides new avenues for enhancing security and making face recognition technologies more reliable.

This manuscript has the follow parts: Section 2 presents work related to the field, and Section 3, features detailed methodology information. Section 4 presents our observation and experimental results, along with technical details. Section 5 describes our implementation in detail. Section 6 presents limitations and future suggestions. In Section 7, we present the conclusions.

## 2. Previous Research

In the last ten years, face PAD approaches have advanced from traditional constructed-characteristic methods to deep learning. Cross-domain PAD has become a recent academic hotspot, prompting the investigation of domain generalization and adaptation strategies to enhance PAD models in unexplored areas  [17]. This section will first discuss the evolution of PAD approaches, then focus on domain generalization and adaptation techniques.

### 2.1. Methods

Face presentation attack detection (PAD) is a crucial task in biometric security that involves distinguishing genuine users from attack presentations (APs) [18]. Due to their cost-effectiveness and ease of implementation, RGB-image-based approaches are widely used in PAD systems [19]. Early PAD methods relied on handcrafted features designed by domain experts, focusing on aspects such as texture evaluation and image quality analysis. However, these approaches were limited by their reliance on prior knowledge about specific attack types and struggled to generalize across various attack techniques.

With the advent of deep learning, PAD systems have evolved significantly. The initial application of deep learning in PAD utilized the VGG-Net feature extraction model, leading to the development of numerous data-driven approaches. The performance of deep-learning-based PAD models depends on several factors, including neural network architectures, optimization strategies, dataset diversity, and annotation accuracy. Simple binary-class neural networks often fail to achieve satisfactory results. To enhance PAD effectiveness, auxiliary learning tasks such as depth estimation, reflection detection, texture mapping, binary mask generation, and adversarial attack modeling have been introduced. Additionally, deep learning models have been applied to extract AP cues from image sub-patches [20]. However, a persistent challenge is the acquisition of diverse attack data samples, as real-world attacks vary unpredictably. To address this, anomaly-detection-based PAD algorithms have been proposed, aiming to detect previously unseen attacks by identifying deviations from learned genuine face patterns. Despite their success in intra-domain testing, current data-driven PAD methods remain limited by the diversity of training data [21] available from source domains. Recent advancements in PAD research have introduced novel techniques to address these challenges. Keyao Wang and colleagues from Baidu Inc. and the University of Chinese Academy of Sciences [22] proposed a multi-domain incremental learning (MDIL) method to enhance face presentation attack detection (PAD). Their approach employs an adaptive domain-specific experts (ADE) framework based on vision transformers, enabling the model to retain discriminative features across multiple domains. Additionally, an asymmetric classifier ensures consistent output distributions, improving generalization. The MDIL method effectively mitigates catastrophic forgetting in PAD by preserving prior domain knowledge, while integrating new domain-specific features. This enhances the model’s robustness against diverse presentation attacks. However, the study did not explicitly outline potential drawbacks, but like most deep-learning-based approaches, its effectiveness may depend on the quality and variety of training data.

Uğur Turhal and colleagues developed a PAD method that extracts multi-level local binary pattern (LBP) [23] features from HSV and Lab color spaces. These features are integrated into a face-weighted multi-color multi-level LBP (FW-MCML-LBP) vector, which is then classified using a support vector machine (SVM). The method demonstrated low error rates on the CASIA and Replay-Attack databases, indicating its efficiency in detecting presentation attacks. The use of multiple color spaces and multi-level texture features enhances its robustness. However, the model’s reliance on color-based texture features may limit its effectiveness under varying lighting conditions or when dealing with grayscale images. Furthermore, its adaptability to unseen attack types remains uncertain.

Weiguo Wan [24] introduced a knowledge-distillation-based approach that integrates convolutional self-attention networks for masked face recognition. A teacher–student framework is employed, where the teacher model guides the student model to focus on unmasked facial regions for enhanced feature extraction. The combination of knowledge distillation and self-attention improves the model’s ability to recognize masked faces, addressing the challenge posed by occlusions. However, the model may require significant computational resources, due to the self-attention mechanism. Further validation is needed to assess its performance across diverse mask types and real-world settings.

Another research study explored a knowledge distillation approach that leverages synthetic data generated through dynamic latent sampling [25] to train face recognition models. The objective is to improve student network performance without extensive reliance on real datasets. The use of synthetic data reduces the dependency on large-scale real-world datasets, potentially lowering data collection costs and addressing privacy concerns. However, the generalization of synthetic data remains a challenge—if the generated samples do not adequately represent real-world variations, the model may struggle in practical applications.

Simone Carta [26] investigated the interpretability of fingerprint PAD systems by analyzing the representativeness of training samples. They employed dimensionality-reduction techniques to visualize sample distributions and assess weaknesses in decision boundaries. The research provided valuable insights into the generalization capabilities of PAD models, emphasizing the importance of diverse and representative training data. While the study enhanced interpretability, it did not propose specific improvements to PAD models. Translating these findings into practical enhancements would require further research.

The field of PAD has transitioned from traditional handcrafted feature extraction methods to deep-learning-driven approaches. While deep learning has significantly improved PAD performance, challenges remain in ensuring robustness against unseen attack types and maintaining adaptability across different domains. Recent studies have introduced innovative solutions such as incremental learning, multi-domain feature extraction, self-attention networks, and knowledge distillation using synthetic data. These advancements have collectively enhanced the generalization and interpretability of PAD systems, laying the foundation for more secure and reliable biometric authentication methods.

### 2.2. Domain Adaptation and Generalization

Domain shift challenges face PAD, seriously affecting the generalization and robustness of models in general. In the past couple of years, domain generalization and domain adaptation approaches have started to appear, among which some methods have been evaluated for improving performance in the target domain [27]. As for domain generalization [28] methods, they suppose that target domain data are not accessible to train the model; therefore, they develop a model that generalizes across multiple source domains. They use generalizable features to extract universal face PAD models. These frameworks perform meta-learning in parallel with numerous scenarios with domain shifts, which are simulated and regularize based on the knowledge of the domain. In addition, even better performance has been achieved in the target domain using domain adaptation techniques [29] when there is a scarcity of training data from the target domain. Many current studies focus on the issue of face PAD through unsupervised domain adaptation, to boost the efficiency of face PAD models using unlabeled data from the target domain. One-class domain adaptation can be employed to improve evaluation of these models in situations where there are changes in the target and source domains [1]. These methods overcome the authentic face samples of the target domain, and our research intends to enhance the execution of face PAD.

Ref. [10] introduced a one-class knowledge distillation approach for face PAD, where a mentor network trained on an extensive dataset containing both genuine and spoofed face images transfers knowledge to a learner network that is trained exclusively on genuine face data. The main objective of this method is to enhance the generalization capabilities of the student network to effectively detect unseen attacks. Unlike conventional PAD models that require extensive labeled attack presentations (APs), this approach treats PAD as an anomaly detection problem, enabling the student network to identify irregularities in facial patterns that could indicate a attack presentation (AP) attempt. By only learning from genuine samples and leveraging the distilled knowledge from the teacher, the student network is expected to recognize deviations that signify presentation attacks, thereby improving detection performance against previously unseen attack types.

Ref. [30] presented Eyepad++, a framework based on knowledge distillation, tailored for simultaneous eye authentication and presentation attack detection using periocular images. Their approach utilizes periocular biometric features to perform authentication, while simultaneously detecting attack presentations (AP). The motivation behind this method is the realization that the eye region provides rich biometric information that is less susceptible to variations in lighting, pose, or occlusion compared to full-face images. The teacher network in Eyepad++ is trained on a broad dataset of periocular images, including both real and spoofed samples, and then transfers its knowledge to a lightweight student network optimized for efficient real-time inference. This method demonstrates the effectiveness of periocular biometrics in PAD, showing that, even in cases where full-face data are unavailable or unreliable, periocular images can serve as a robust biometric modality for secure authentication.

More recently, ref. [31] employed diffusion models alongside knowledge distillation to enhance document PAD. Their work explored the application of generative models, specifically diffusion models, to create highly realistic synthetic attack samples. By integrating these diffusion-generated attack variations into a knowledge distillation framework, their approach enhances the student network’s ability to recognize document-based attack presentations (APs), such as printed or digital manipulations of identification documents. The primary contribution of this study lies in demonstrating how generative AI can be leveraged to strengthen PAD systems by synthesizing diverse attack scenarios that may not have been present in the training data. This strategy significantly improves the generalizability of PAD models in document authentication tasks, reducing their reliance on manually curated attack datasets.

While these works provided valuable insights into the use of knowledge distillation for PAD, our approach diverges in several crucial aspects. Firstly, we enhance the teacher–student learning paradigm by incorporating a structured adversarial training framework, which exposes the student network to adversarially generated attack samples during training. This additional training mechanism improves the student network’s adaptability, enabling it to better generalize to novel attack types beyond those included in the training set. Unlike [10], which concentrated on single-class learning, our approach actively trains the learner network with adversarial variations, enhancing its capability to differentiate between authentic and attack samples with greater accuracy.

Secondly, unlike prior studies that primarily focused on document-based PAD [10] or periocular biometrics [30], our model introduces the integration of facial expressions and dynamic backdrops as additional discriminative features. Incorporating facial expressions improves the model’s capacity to distinguish between genuine and fake faces by utilizing subtle movements and muscle patterns that are challenging to imitate in attack presentations (APs). Similarly, incorporating dynamic backgrounds ensures that our PAD model considers contextual cues beyond the facial region, further strengthening its resistance to sophisticated attack vectors such as deepfake-based or 3D-mask-based attack presentations (APs).

Finally, we propose a sparse training mechanism for the student network, which optimizes computational efficiency, while maintaining high detection performance. By selectively retaining only the most relevant parameters within the student network, our approach reduces the model’s size and computational overhead, making it more suitable for real-time applications. This contrasts with diffusion-based approaches like [10,30], which, while effective, can be computationally expensive and challenging to deploy in resource-constrained environments. Our sparse learning strategy ensures that the student network remains lightweight and efficient, without compromising detection accuracy, making it more adaptable for deployment in real-world biometric authentication systems.

## 3. Methodology

Our methodology addresses the growing complexity of biometric security threats by enhancing face presentation attack detection (PAD) accuracy. At its core, our approach integrates a teacher–student learning framework with advanced PAD techniques to improve adaptability and real-world performance. Traditional PAD methods, while effective against known attack presentation (AP) tactics, often struggle with novel and sophisticated attack strategies. To overcome this limitation, our model is designed to enhance generalization and robustness, ensuring stronger defense against emerging threats.

**Figure 2:**
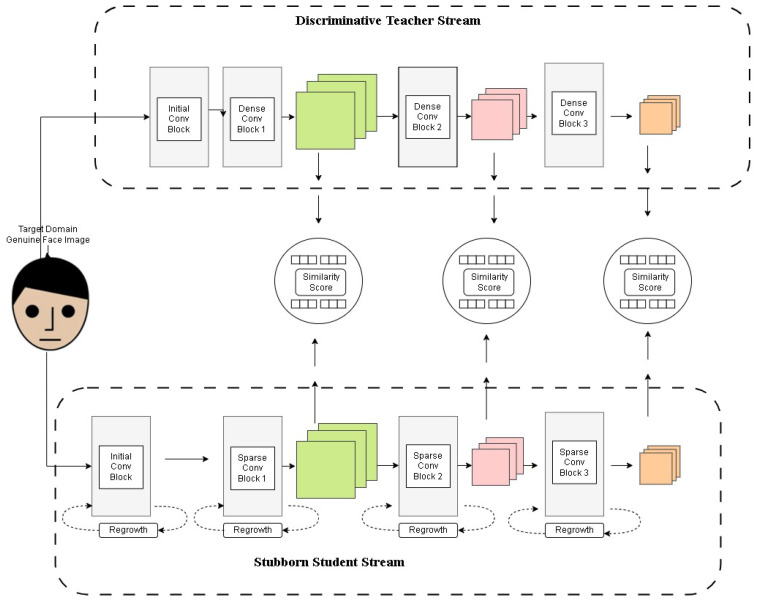
The diagram illustrates the Stubborn Student (SS) network training process. Real images from the semantic domain are passed through SS network and DT network, extracting key features. The similarity between extracted features refines the SS network, optimizing its parameter density for better efficiency.

In our system, a mentor network focuses on a diverse set of genuine and attack samples and transfers its knowledge to a learner network. This knowledge distillation process enables the student network to differentiate between real and spoofed facial presentations with high precision, even when encountering unseen attack types shown in Figure 2. Unlike conventional PAD methods, which rely heavily on dataset-specific features, our approach enhances adaptability by incorporating deep-learning-based feature analysis and adversarial training to improve detection accuracy.

By shifting from static, dataset-dependent PAD models to a more dynamic and learning-driven system, our method establishes a new benchmark in biometric security. This adaptability ensures that face recognition technologies remain resilient and trustworthy, even as cyber threats evolve in complexity.

### 3.1. Innovative Integration of the Teacher–Student Learning Framework

To enhance face presentation attack detection (PAD), we put-forward a mentor–learner studying framework that effectively transfers knowledge from a well-trained teacher network to a more adaptable student network. This approach addresses key challenges in PAD by ensuring the student network can generalize well to unseen attack types, while remaining computationally efficient.

At the core of this framework, the teacher network is trained on a diverse dataset that includes a wide range of attack presentation (AP) attacks, from sophisticated 3D mask attacks to photo and video-based spoofs. By learning intricate patterns that differentiate genuine faces from attacks, the teacher network develops a robust feature-extraction capability, ensuring accurate classification across different attack scenarios. This knowledge distillation process enables the student network to inherit critical decision-making patterns from the teacher, while remaining lightweight and adaptable.

**Figure 3:**
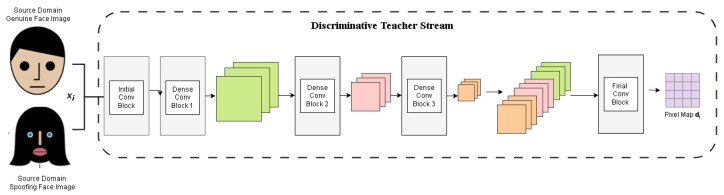
The diagram depicts the DT network training with source domain data. Each face image $x_{i}$ is processed through multiple convolutional layers, extracting features $f_{i}^{1}$, $f_{i}^{2}$, and $f_{i}^{3}$. These features are then combined to estimate a pixel map $d_{i}$ for enhanced spoof detection.

The teacher network is designed to prioritize and extract essential features that distinguish genuine facial attributes from spoofed ones. It employs deep convolutional layers to analyze biometric data and identify subtle texture, motion, and depth cues indicative of attack presentations (APs) as shown in Figure 3. The extracted feature representations serve as the foundation for the student network, allowing it to make informed decisions with minimal training data.

Unlike the mentor network, the learner network focuses on refining its understanding of genuine face representations within specific target domains. Instead of directly learning from attack samples, the student absorbs the distilled knowledge provided by the teacher network, allowing it to recognize genuine faces with high precision. This strategic specialization makes the student network more efficient and adaptable, particularly in scenarios where attack presentation (AP) attacks continuously evolve.

The training process is carefully structured to expose the student network to a curated subset of data used to train the mentor network. This subset is optimized to highlight the subtle, yet distinctive cues of genuine faces, ensuring the student network remains focused on authenticity detection, without unnecessary complexity. The learning process is reinforced through multilevel similarity measurement, where the student progressively refines its feature representation to align closely with the teacher’s outputs, as detailed in Algorithm 1.
**Algorithm 1:** Algorithm for Inequitable Teacher Network Training**Input**: Veritable face images from ($B_{src}$), Attack Presentations (AP) samples from ($B_{attack}$), Maximum training Iteration $K_{1}$, Hyper parameters for training, learning Rate $\alpha_{1}$, Batch size $N_{1}$**Results**: DT parameters $\theta_{DT}$1.**Initialize** Model parameters as, →$\theta_{IT}$ for IT and $\theta_{FCB}$ for FCB;2.**For** i=1 **to** $i_{1}$ **do**3.      specimen $n_{1}$, specimen $x_{i}$ with stamps $y_{i}$ from $B_{src}$4.      Extracting feature $f_{i}^{1},f_{i}^{2},f_{i}^{3}$ by encoding $xi\;with\;\theta_{IT}$5.      Pixel map prediction $p_{i}$ by processing $f_{i}^{1},f_{i}^{2},f_{i}^{3}$ with $\theta_{FCB}$6.      Comp $L_{IT}$ with $p_{i}$ and $b_{i}$ like in Equation (1)7.      Update $\theta_{IT}\;and\;\theta_{FCB}\;with\;L_{DT},\alpha_{1}$.8.**end for loop**9.**Result:** return $\theta_{I}T$;

Our teacher–student framework represents a significant leap in PAD methodologies by ensuring that face recognition systems remain adaptive, scalable, and resistant to emerging attack types. By enabling efficient knowledge transfer and real-time generalization, this framework not only enhances current PAD systems but also lays the foundation for future advancements in biometric security.

### 3.2. Advanced PAD Methodologies

To improve the precision and resilience of face presentation attack detection (PAD), cutting-edge deep learning techniques are incorporated into our system. By leveraging leading edge techniques, the model attains improved feature extraction and attack presentation (AP) sensing through a combination of Convolutional Neural Networks (CNNs), activation functions, pooling mechanisms, and optimized loss functions. These components collectively enable a more comprehensive analysis of facial textures, expressions, and biometric cues, effectively distinguishing genuine faces from spoofed presentations.

Convolutional Neural Networks (CNNs) serve as the backbone of our PAD system, demonstrating an exceptional capability for processing facial image data. CNNs extract hierarchical feature representations through multiple layers of convolution operations, which help detect texture inconsistencies and attack presentation (AP) artifacts. The convolution operation is mathematically defined as

(1) $$F_{ab}=\sum_{i}\sum_{j}I_{\left(a+i)(b+j\right)}K_{ij}$$

where $F_{ab}$ represents the output mapping of features, *I* is the input image, and *K* is convolutional quintessence. This operation enables the network to capture spatial hierarchies in the input facial data, allowing it to identify distinct patterns indicative of attack presentations (APs).

Following convolution, the activation function introduces a roundabout into network, allowing it to learn intricate patterns that differentiate real and fake facial presentations. We employ the Rectified Linear Unit (ReLU) activation function, which is mathematically defined as follows:

(2) ReLU(x)=max(0,x)#xismaxlimit

*ReLU* ensures that only positive values are retained in the feature map, effectively filtering out irrelevant information and tweaking the network’s ability to focus on important facial details. This improves the model’s ability to pick out genuine from fake faces, even with challenging lighting and pose variations.

To manage computational complexity and retain essential information, pooling layers are applied to reduce feature map dimensions, while preserving key patterns. Max pooling is a widely used technique in our model, defined as:

(3) $$P_{ij}=\max_{a,b\in M_{ij}}F_{ab}$$

where $P_{ij}$ is the pooled feature map and $M_{ij}$ represents a local region in the input feature map. This operation ensures that only the most significant features are retained, making the network resilient to spatial variations in the input images.

The effectiveness of the CNN model is further enhanced through loss function optimization, which ensures accurate classification of genuine and spoofed facial images. We use a cross-entropy loss function, widely adopted for classification tasks, defined as

(4) $$L\left(z,\hat{z}\right)=-\sum_{n}z_{o,n}\log\left({\hat{z}}_{o,n}\right)$$

where *z* represents the true class label, $\hat{z}$ denotes the predicted probability, and the summation runs over all classes *n*. This function measures the deviation between the speculated and true labels, guiding the model to refine its predictions through backpropagation and gradient descent optimization. Our deep-learning-based PAD model undergoes end-to-end training, where

- Convolutional layers extract low-level and high-level facial features.
- Activation functions introduce non-linearity to capture complex patterns.
- Pooling layers reduce the feature map size, retaining key information.
- The loss function optimizes the network performance through backpropagation.

By integrating these advanced deep learning methodologies, our approach significantly improves PAD accuracy, making it more robust to varied lighting conditions, pose variations, and unseen attack types.

### 3.3. Pertinacious Student Network Training

To further enhance the strength and adaptability of the PAD system, we introduce the Pertinacious Student (PS) network. The PS network is designed to refine its understanding of genuine facial representations by learning from the Teacher Network (IT), while maintaining computational efficiency. The key objective of the PS network is to ensure that its feature representations closely align with those extracted by the IT network, thereby enabling it to generalize well to unseen target domains.

During training, real face photos from the target domain are input to both the mentor network (IT) and learner network (PS) for feature extraction. The feature representations extracted from both networks are compared using cosine similarity, allowing the PS network to iteratively adjust its parameters to match the teacher’s learned representations.

**Figure 4:**
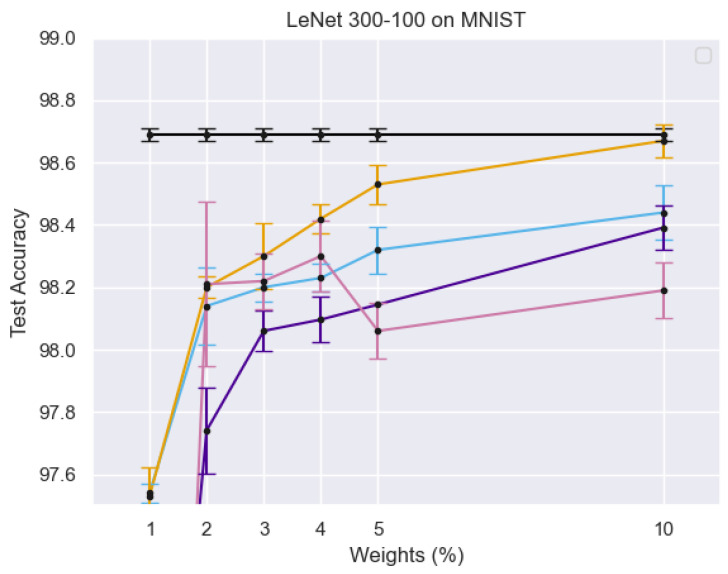
Student network training process: Real face images from the target domain are passed through both teacher and student networks, with multi-level similarity computation used for optimization.

Figure 4 illustrates the training process of the Student Network, where real face images from the target domain are passed through both the teacher and student networks. The different curves in the graph represent test accuracy trends under varying percentages of active weights, showing the impact of sparse training on model performance. The error bars indicate the variance in accuracy, demonstrating the robustness of the PS network. The results suggest that using a limited number of active weights still achieves competitive accuracy, reinforcing the efficiency of the sparse training strategy.

**Figure 5:**
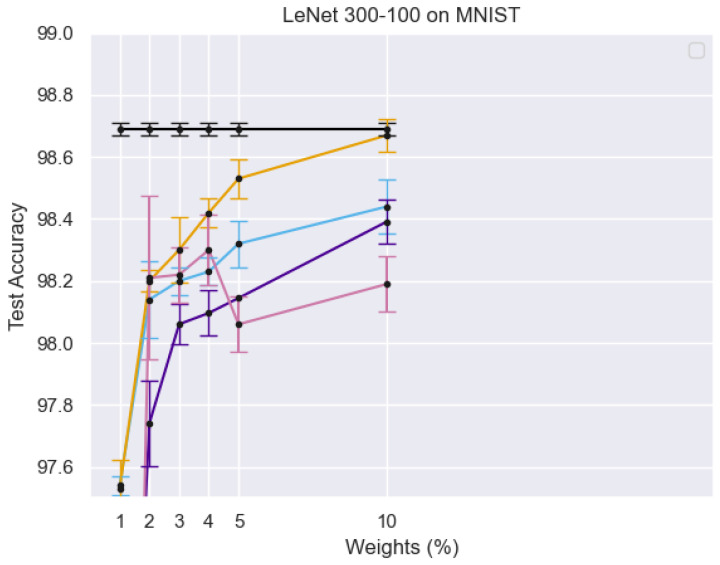
The diagram illustrates the training process of the PS network, where target domain face images are input into both the teacher and student networks. Multi-level similarity is computed to optimize the PS network through sparse training.

Figure 5 illustrates the training process of the PS network, where target domain face images are processed within both the teacher and student networks. Multi-level similarity is computed to optimize the PS network using sparse training. *X*-axis (Weights %): Represents the percentage of active parameters in the sparse PS network. *Y*-axis (Test Accuracy): Measures the model’s performance on the MNIST dataset. Different Colored Lines: Represent different experimental configurations, highlighting variations in model architectures, training strategies, or sparsity levels. Black Line with Error Bars: Likely represents a baseline model or fully dense network for comparison. Other Lines: Show how test accuracy improves or fluctuates as the percentage of active weights increases, demonstrating the impact of sparse training on classification performance.

The feature representations generated by the IT and PS networks are given by

(5) $$f_{i}^{1},f_{i}^{2},f_{i}^{3},\;\mathrm{and}\;f_{i}^{\prime 1},f_{i}^{\prime 2},f_{i}^{\prime 3}$$

where $f_{i}^{1},f_{i}^{2},f_{i}^{3}$ are feature representations from the teacher network (IT), and $f_{i}^{\prime 1},f_{i}^{\prime 2},f_{i}^{\prime 3}$ are feature representations from the student network (PS).

A cosine similarity function is used to evaluate the sequence between these feature representations, as defined in Equation (6):

(6) $$S\left(f,f^{\prime }\right)=1-\frac{\left(f\cdot f^{\prime }\right)}{\left(\left|f\right|\left|f^{\prime }\right|\right)}$$

where *f* and $f^{\prime }$ represent feature vectors. The Learning Similarity (LS_S_) function, which serves as the optimization objective, is defined as

(7) $$L_{PS}=\frac{1}{N_{2}}\sum_{i=1}^{N_{2}}\lambda_{1}S\left(f_{i}^{1},f_{i}^{\prime 1}\right)+\lambda_{2}S\left(f_{i}^{2},f_{i}^{\prime 2}\right)+\lambda_{3}S\left(f_{i}^{3},f_{i}^{\prime 3}\right)$$

where $\lambda_{1},\lambda_{2},\lambda_{3}$ are weighting factors controlling the contribution of different feature levels.

The learner network optimization function is formulated as follows:

(8) $$\arg\min_{\theta_{PS}}E_{z∼B_{trg}}L_{PS}\left(z|\;\theta_{IT},\theta_{PS}\right)$$

where $\theta_{IT}$ and $\theta_{PS}$ are parameters of the mentor and learner networks, respectively.

**Figure 6:**
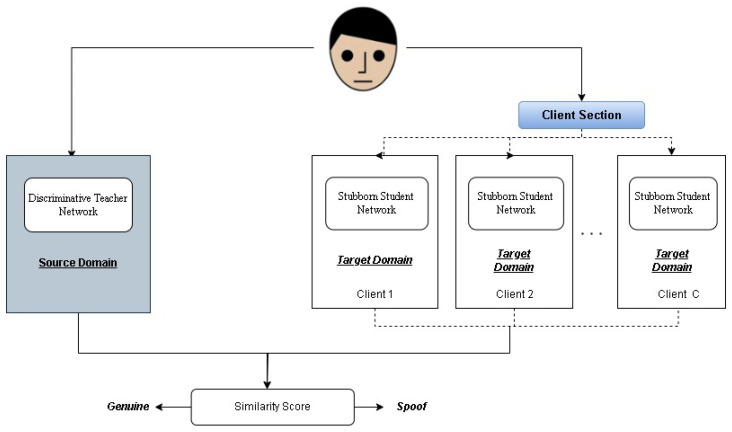
When client-specific one-class domain adaptation is performed, our model employs a DT network and multiple SS networks, each tailored for a specific client. Face images are tested through both the IT network and the respective SS network for client-specific inference.

Employing multiple PS networks increases the total number of features in the system, which poses a challenge in terms of computational efficiency. To mitigate this, we introduce a sparse training technique, where the PS network utilizes sparse convolution kernels, ensuring that only a selected percentage (s%) of the parameters are active. This client and source domain setup is shown in Figure 6.

The determination of active and inactive parameters in the convolution layers is defined as follows:

(9) $$X_{j}=\left\{i∥u_{j,i}|\geq \tau_{j}\right\},\;Y_{j}=\left\{i∥u_{j,i}|<\tau_{mj}\right\}$$

where $X_{j}$ represents active parameters, $Y_{j}$ denotes inactive parameters, $u_{j,i}$ is the initial indicator, and $\tau_{j}$ is the threshold.

To maintain network efficiency, pruning is applied using Equation (10), where a portion of the least important parameters is removed:

(10) $$P_{j}=\left\{i|i\in X_{j},\left|\omega_{j,i}\right|<\tau_{j}^{p}\right\}$$

New parameters are grown through an adaptive regrowth mechanism, defined as

(11) $$p_{j,i}=\beta_{1}p_{j,i}^{\prime }+\left(1-\beta_{1}\right)\frac{\partial L_{PS}}{\partial \omega_{j,i}}$$

(12) $$q_{j,i}=\beta_{2}q_{j,i}^{\prime }+\left(1-\beta_{2}\right){\left(\frac{\partial L_{PS}}{\partial \omega_{j,i}}\right)}^{2}$$

where $\beta_{1}$ and $\beta_{2}$ are smoothing factors. The updated growth selection is defined in Equation (13):

(13) $$G_{j}=\left\{i|i\;\;\;\epsilon \;\;Y_{j},\;\left|\mu_{j,i}\right|\geq \tau_{j}^{g}\right\}$$

To further enhance the PS network’s resilience, we incorporate adversarial training and data augmentation. The adversarial loss function, optimizing detection against adversarial samples, is defined as

(14) $$L_{adv}=-\sum y*\log\left(\mathrm{pred}\right)$$

Random cropping, rotation, flipping, and color jittering improve the student network’s generalization ability.

The final training objective of the PS network combines both adversarial and supervised loss functions:

(15) $$L_{total}=\lambda_{adv}*L_{adv}+\lambda_{sup}*L_{sup}$$

where $\lambda_{adv}$ and $\lambda_{sup}$ balance the contributions of the adversarial and supervised loss The overall pertinacious student network training algorithm shown in Algorithm 2.

### 3.4. Classification Techniques

Traditional classification methods often focus solely on facial appearance, neglecting contextual factors such as facial expressions and background information. However, these factors can significantly impact classification performance, especially for challenging samples.

We employ facial expression analysis to enhance the classification performance by capturing subtle variations in eye movements, eyebrow positioning, and mouth shape. This allows our model to differentiate natural expressions from artificially manipulated ones, a key indicator of attack presentation (AP) attacks. Our method builds upon existing facial analysis techniques, such as those in [32], where facial micro-movements were used to improve biometric authentication.

Background cues often provide additional discriminatory information in PAD. Object detection and scene recognition techniques are integrated into our model to analyze the surrounding environment of an input face. This helps in detecting inconsistencies in the scene, which are common in photo, video, and deepfake-based attacks.

Given an input face image, our model performs feature extraction using a deep convolutional network, followed by a classification stage. The extracted features are processed using a fully connected layer and a softmax function to classify the image as genuine or attack:

(16) P(y|x)=softmax(W·F+b)

where *W* represents the weight matrix, *F* is the feature vector extract, and *b* is a bias term.

To further increase the effectiveness of our PAD model, we employ adversarial training, where the model is exposed to synthetically generated attack samples. Instead of detailing the adversarial loss, we refer the reader to prior works on adversarial learning.

**Figure 7:**
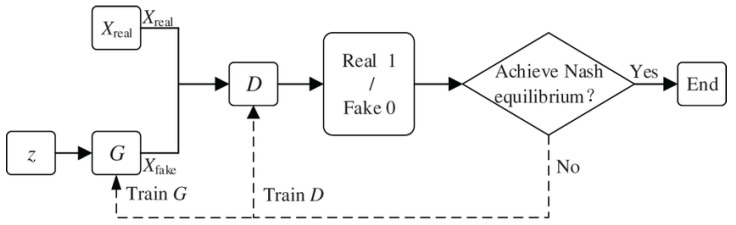
Basic structure of a generative adversarial network (GAN) [33].

We utilize a GAN network shown in Figure 7, where

- The Generator produces synthetic attack samples.
- The Discriminator attempts to pick out real from fake samples.

**Algorithm 2:** Pertinacious Student Network Training Algorithm
**Input**: Genuine face images from $D_{tgt}$, IT parameters $\theta_{DT}$, sparse selection (SS) density s%, initial regrowth rate r%, learning rate $\alpha_{2}$, maximum training iterations $K_{2}$, batch size $N_{2}$, and regrowth period *T***Result**: SS parameters $\theta_{SS}$

1.**Initialize** model parameters $\theta_{SS}$;2.For each convolutional layer, determine the parameter count $l_{m}$ for the *m*-th layer;3.Identify indices $A_{m}$ for active parameters totaling $l_{m}\cdot s\%$ as per Equation (6), and $B_{m}$ for inactive parameters totaling $l_{m}\cdot \left(1-s\%\right)$ as per Equation (7);4.Set parameters $\omega_{m,n}=0$ for all $n\in B_{m}$;5.**For** K=1 **to** $K_{2}$ **do**(a)Sample $N_{2}$ examples $z_{i}$ from $D_{tgt}$;(b)Extract features $f_{1i}$, $f_{2i}$, $f_{3i}$ using $\theta_{DT}$, and features $f_{\bar{1}i}$, $f_{\bar{2}i}$, $f_{\bar{3}i}$ using $\theta_{SS}$;(c)Calculate $L_{SS}$ with $f_{1i}$, $f_{2i}$, $f_{3i}$, $f_{\bar{1}i}$, $f_{\bar{2}i}$, $f_{\bar{3}i}$ as shown in Equation (4);(d)Update active parameters in $\theta_{SS}$ using $L_{SS}$, $\alpha_{2}$, and $A_{m}$;(e)Adjust regrowth rate r% using cosine decay;(f)**If** KmodT=0 **then**i.Count active parameters $|A_{m}|$;ii.Identify indices $P_{m}$ for the $|A_{m}|\cdot r\%$ parameters to be pruned as per Equation (8);iii.Update active and inactive parameter indices: $A_{m}\leftarrow A_{m}-P_{m}$, $B_{m}\leftarrow B_{m}\cup P_{m}$;iv.Identify indices $G_{m}$ for the $|A_{m}|\cdot r\%$ parameters to be grown as per Equations (9)–(12);v.Update active and inactive parameter indices: $A_{m}\leftarrow A_{m}\cup G_{m}$, $B_{m}\leftarrow B_{m}-G_{m}$;6.Compute similarity between teacher and student representations using the defined measurement;7.Calculate loss $L_{PS}$ based on the weighted similarity sum at different feature levels;8.Update Pertinacious Student (PS) network weights using gradient descent or an appropriate optimizer, minimizing $L_{PS}$ with respect to student network parameters ($\theta_{PS}$);9.Apply sparse training to adjust the non-zero parameter density in the PS network based on indicator magnitudes;10.Perform regular parameter regrowth by pruning inactive parameters and growing new active ones based on their potential to reduce $L_{PS}$;
**Result**: Return $\theta_{PS}$


During training, the teacher network acts as the discriminator, while the student network learns adversarial patterns. The adversarial loss function follows the standard GAN formulation, as in Equation (16). This ensures that the student network learns more generalized attack features, improving its ability to detect unseen attacks.

The use of synthetic data in our model raises concerns regarding privacy and ethical implications, particularly the risk of misuse and data exposure. To mitigate these risks, we propose incorporating differential privacy techniques in synthetic data generation to prevent leakage of sensitive information. Additionally, access control mechanisms, including encryption and authentication protocols, can help regulate access to generated data. We also implement adversarial training techniques to minimize unintended data disclosure and enhance privacy protection. Bias in attack sample generation is another critical concern, and to address this, we ensure the use of balanced datasets that provide an equal representation of different demographic groups. Furthermore, fairness-aware training techniques and fairness evaluation metrics such as disparate impact and equalized odds are employed to mitigate algorithmic bias and ensure unbiased classification.

### 3.5. Network Architecture and Implementation Details

Our model consists of a deep convolutional neural network (CNN) with the following architecture:

- Input Layer: Takes face images of size 224 × 224 pixels.
- Convolutional Layers (5 layers): Extracts spatial features from facial images.
- Batch Normalization and ReLU Activations: Ensures stable training and non-linearity.
- Pooling Layers (2 layers): Reduces dimensionality, while preserving key facial features.
- Fully Connected Layer (1 layer): Maps features to classification scores.
- Softmax Output Layer: Classifies the image as real or attack.

Our model was trained using PyTorch, and the following hyperparameters were used:

- Batch size: 32
- Learning rate: 0.0001 (using Adam optimizer)
- Number of epochs: 50
- Pretrained weights: The model was initialized with weights from ResNet-50, followed by fine-tuning.

Before classification, face images were cropped using MTCNN (Multi-task Cascaded Convolutional Networks) to ensure accurate face localization. The cropped face was then resized to 224 × 224 pixels before being passed through the neural network.

During inference, the trained model evaluates input face images using both the mentor and learner networks. The teacher network extracts multi-level feature representations, which are processed using Equation (17):

(17) $$\xi_{t}=\frac{1}{3}\left(S\left(f_{t}^{1},f_{t}^{\prime 1}\right)+S\left(f_{t}^{2},f_{t}^{\prime 2}\right)+S\left(f_{t}^{3},f_{t}^{\prime 3}\right)\right)$$

where $S(f,f^{\prime })$ computes the cosine similarity between the teacher and student network features.

The final classification decision is based on a threshold δ:

(18) $$I_{T}=\left\{\begin{array}{cc} \mathrm{genuine}, & \xi_{t}<\delta \\ \mathrm{attack}, & \xi_{t}\geq \delta \end{array}\right.$$

To further improve prediction accuracy, we use

- Ensemble Learning: Aggregates outputs from the teacher and student networks.
- Fine-Tuning: Adjusts the model for target-specific datasets.
- Transfer Learning: Adapts the model to new attack variations with minimal re-training.

## 4. Observations and Experimental Results

To form an opinion of the effectiveness of our present model, in general and in client-specific domain adaptation scenarios, we conducted experiments using multiple protocols. These protocols depicted synthetic real-world settings, ensuring that the model maintained a high accuracy and adaptability across different PAD environments. We evaluated the model’s performance using cross-domain face PAD datasets, including IDIAP REPLAY-ATTACK [20], CASIA-FASD [34], MSU-MFSD [35], NTU ROSE-YOUTU [36], and OULU-NPU [37].

Our approach introduces computational overheads due to the incorporation of multiple facial expression variations and diverse backdrops, which increase the processing power required for feature extraction and classification. This added complexity can impact inference speed, particularly on resource-constrained devices. To address these challenges, we discussed various optimizations, including model compression through pruning and quantization to reduce the model size, parallel processing leveraging GPU acceleration, and the use of lightweight data augmentation techniques to balance computational costs and data diversity. Furthermore, we explored the potential of edge AI models to ensure efficient deployment on resource-limited devices, without compromising accuracy.

To ensure a standardized evaluation of face presentation attack detection (PAD) performance, we used the internationally recognized ISO/IEC 30107-3 [38] standard for biometric PAD appraisal. The following key performance metrics were considered:

- Attack Presentation Classification Error Rate (APCER): Measures the ratio of attack presentations incorrectly classified as genuine.
- Bona Fide Presentation Classification Error Rate (BPCER): Measures the ratio of Bona Fide Presentations (BPs) incorrectly classified as attacks.
- Detection Error Tradeoff (DET) Curves: Visualize the tradeoff between APCER and BPCER, helping in performance comparisons.

### 4.1. General One-Class Domain Adaptation

We tested our face PAD system under one-class domain adaptation using the CIMN One-Class Domain Adaptation protocol, inspired by prior domain adaptation methods mentioned in Table 1. The experiment followed these steps:

- Each dataset was treated as a separate domain, serving as a source or target domain in different experimental settings.
- The model was trained with full source domain training data and genuine face data from the target domain’s training set.
- Performance was assessed using the target domain’s test set, evaluating the model’s generalization ability.
- Comparisons wee made against the OULU One-Class Adaptation Structure A protocol from recent studies, to ensure a standardized evaluation.

### 4.2. Client-Specific Domain Adaptation

Our model’s quality in client-specific one-class domain adaptation was further evaluated using the CIM-N Client-Specific One-Class Domain Adaptation protocol. This setup enhanced the model performance for each target client by using limited authentic face data.

The experiment setup included

- Ten clients selected from the NTU ROSE-YOUTU dataset, each representing a unique target domain.
- Each client had 50 real-life face videos and 110 attack videos.
- Twenty-five real face videos sample and 110 attack videos were used as the test set.
- One frame per client from the remaining 25 authentic videos was used as training data.

Source Domains: CASIA-FASD (C), IDIAP REPLAY-ATTACK (I), and MSU-MFSD (M). The experiment was conducted under three sub-protocols: C-N-CS, I-N-CS, and M-N-CS, evaluating 10 client-specific tasks per protocol as sown in Table 2.

### 4.3. Comparison with Conventional Methods

To validate the performance of our presented system, we implemented several conventional PAD techniques in one-class domain adaptation. These included

**Figure 8:**
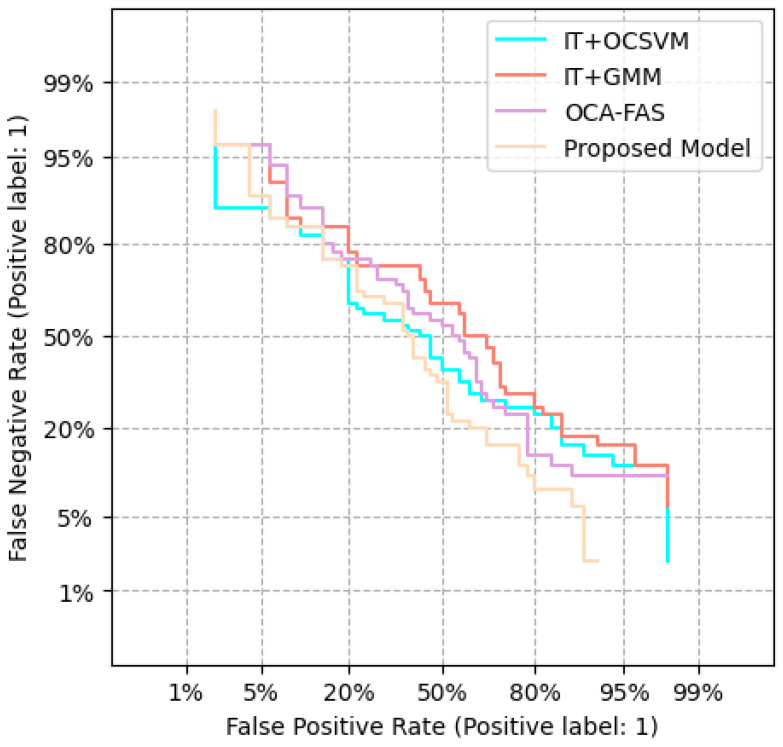
Performance comparison of our proposed model against conventional PAD methods.

- Depth Regression Network (DT): Used as the backbone method, trained on the source domain data shown in Figure 8.
- IT + OCSVM: One-Class Support Vector Machine classifier trained using features from the pre-trained IT model.
- IT + GMM: Gaussian Mixture Model trained using IT-extracted features, commonly used for one-class classification.
- OCA-FAS: A recent one-class adaptation method for face PAD.
- Unsupervised Domain Adaptation Methods: Comparisons were made against KSA, ADA, UDA, and USDAN-Un.

### 4.4. Key Observations

- Our model consistently outperformed the existing PAD methods in general, one-class, and client-specific domain adaptation.
- APCER and BPCER values were significantly lower, ensuring fewer false positives and negatives.
- The introduction of facial expression-based classification and background analysis contributed to improved cross-domain generalization.
- DET curves confirm that our model maintained better performance trade-offs compared to the conventional methods.

## 5. Details of Implementation

To ensure a fair evaluation, we followed a standardized preprocessing pipeline across all datasets. The implementation details, including data preprocessing, model training, and evaluation settings, are discussed below.

We adopted a consistent preprocessing approach for the five datasets used in this study: CASIA-FASD [34], IDIAP REPLAY-ATTACK [20], MSU-MFSD [35], NTU ROSE-YOUTU [21], and OULU-NPU [37]. The data preprocessing steps were as follows:

- Frame Sampling: Unlike previous works that randomly selected 50 frames per video, we followed the data selection protocol of [10], uniformly selecting 25 frames per video. This ensured comparability with standard PAD methods.
- Facial Detection and Alignment: We used the dlib library for face detection and alignment, ensuring consistent cropping and frontal alignment across datasets.
- Facial Resizing: All face images were resized to 128 × 128 pixels, maintaining consistency and reducing computational overhead.

To validate the impact of frame selection, we compared results using 50 random frames and 25 uniformly selected frames. The results indicated minimal performance differences, supporting our decision to adopt the standardized 25-frame selection.

The final number of images obtained per dataset after preprocessing were CASIA-FASD—30,000, IDIAP REPLAY-ATTACK—58,400, MSU-MFSD—13,700, NTU ROSE-YOUTU—161,950, and OULU-NPU—246,750.

We used the Adam optimizer to train both the DT network and SS network. The IT network was trained over 8400 renewals with a small batch size of 30 and learning rate of $10^{-4}$. The SS network was trained over 1500 iterations with a mini-batch size of 25 and the same learning rate. In our framework, we set the weights in Equation (4) to be equal, each with a value of 0.33. For tests with SS densities of 10% and 1%, we set the regrowth period *T* to 60 and the initial regrowth rate r% to 50% and 20%, respectively, using cosine decay to adjust the regrowth rate.

We used PyTorch version 1.7.0 to develop our suggested framework as well as the baseline techniques, ensuring compatibility and leveraging the deep learning framework’s capabilities.

### 5.1. General Domain Adaptation Experiments

#### 5.1.1. Experiment with CIMN-OCDA

We tested the efficiency of our suggested methodology for face presentation attack detection (PAD) using the CIMN-OCDA protocol. Our approach was compared to baseline methods, all utilizing the same IT model as feature extractor. The student (PS) model’s density was fixed at 10%. We evaluated the cross-dataset achievements of the face PAD techniques with two experimental setting: ideal, and demanding. The experimental results for the ideal settings are shown in Table 3.

The Half Total Error Rate (HTER) performance was directly computed on the target dataset’s test set using the optimal threshold in the ideal environment. Table 3 shows the results for this optimal configuration. In the demanding setting, the HTER was computed at a threshold selected on a validation set, with the False Rejection Rate (FRR) set to 10%. For trials using dataset I as the target, authentic face samples from the development set served as the evidence set. For datasets C, M, and N, which did not have a development set, we used 20% of the real face samples from the target dataset’s training set as a validation set, as shown in Table 4. Table 5 presents the outcomes for the demanding situation.

Our proposed methodology consistently achieved a significant reduction in HTER across the various tasks compared to the DT method Table 6. The average HTER reduction exceeded 10%, demonstrating the effectiveness of our strategy in improving face PAD performance with a small number of authentic face samples from the target domain. Furthermore, our technique outperformed the streamline methods using 1C domain adaptation, achieving the best performance in the ideal and challenging trial scenarios shown in Table 7.

**Figure 9:**
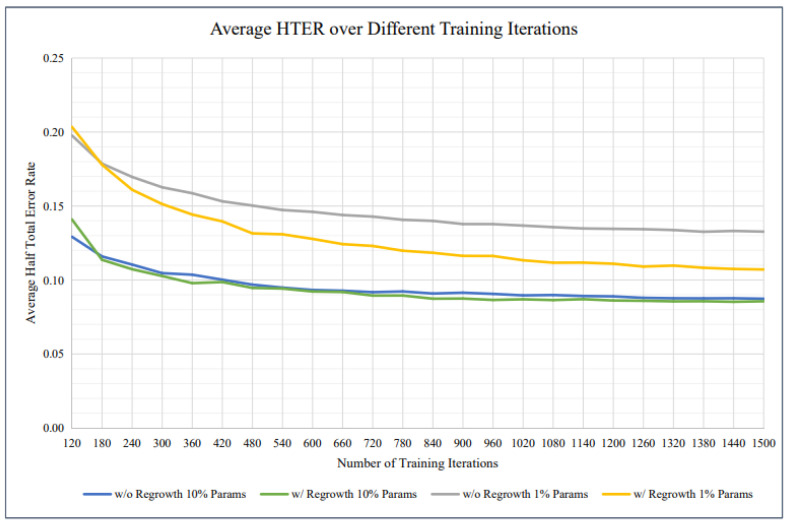
The average HTER of our suggested approach is shown in the graph for different training iterations.

In Figure 9, apresents a comparative analysis of average Half Total Error Rate (HTER) across different training iterations for models trained with and without parameter regrowth. The graph includes four curves representing different conditions: models trained without regrowth using 10% parameters (blue line), with regrowth using 10% parameters (green line), without regrowth using 1% parameters (gray line), and with regrowth using 1% parameters (yellow line). The trends indicate that models incorporating parameter regrowth generally achieve lower HTER, demonstrating improved performance. Additionally, models trained with 10% parameters consistently outperform those using only 1% parameters. This comparison highlights the effectiveness of regrowth strategies in enhancing model training efficiency and accuracy.

#### 5.1.2. Experiments on OULU-OCA-SA

**Figure 10:**
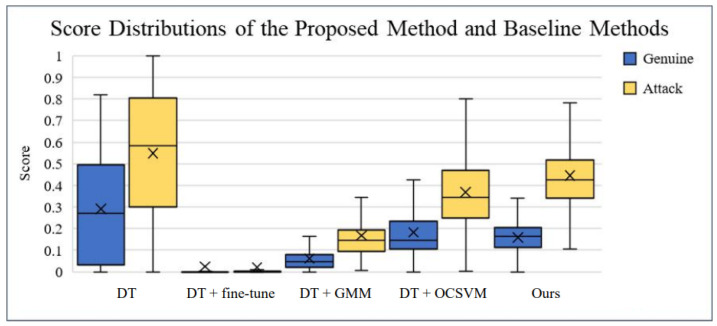
The graphic depicts the score distributions of the various approaches for C-N-CS job 1. The spatial distributions of the actual face and attack samples are depicted in blue and yellow boxes, respectively. The graph illustrates that our method had a wider separation gap than the baseline methods.

For our proposed technique against OCA-FAS using the OULU-OCA-SA protocol, a recent method for PAD, we tested our approach on Protocol 3 in OULU-OCA-SA, setting the density of the Student (SS) model to 10%. The experimental results for Protocol 3, as shown in Table 4, revealed that our technique significantly outperformed both DTN and OCA-FAS. Our approach achieved an AUC of 99.98% and an Average Classification Error Rate (ACER) of 0.08%. Incorporating genuine face samples from the targeted domain for domain adaptation led to a 3.49% reduction in ACER and a 1.46% improvement in AUC compared to our baseline technique shown in Figure 10.

### 5.2. Client-Specific Domain Adaptation Experiments

We conducted experiments in a general one-class domain adaptation setting, demonstrating the strong effectiveness of our proposed method for improving face presentation attack detection (PAD) performance using only a few genuine face images from specific target clients shown in Table 8.

**Figure 11:**
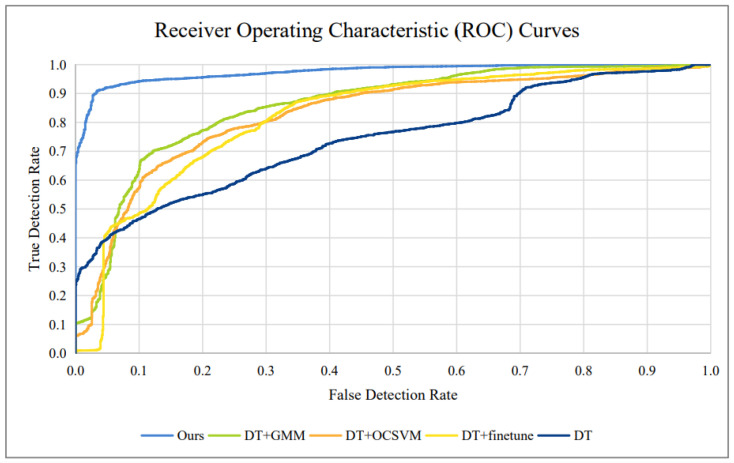
Displays ROC curves for various methods applied to C-N-CS task 1.

Furthermore, Figure 11 depicts the Receiver Operating Characteristic (ROC) curves of our presented approach and baseline methods on job 1. For various False Detection Rate (FDR) situations, the True Detection Rate of our technique outperformed the TDRs of the baseline methods. The benefit of our strategy was especially noticeable for low FDR thresholds.

To evaluate the robustness of our approach across cultural and ethnic contexts, we conducted tests on datasets containing diverse ethnic groups and cultural backgrounds. Our findings suggested that variations in skin tone, facial structure, and cultural expressions could influence model performance. While our method demonstrated strong generalization across different ethnicities, minor discrepancies in accuracy were observed due to dataset imbalances. To enhance fairness and inclusivity, we propose expanding the training data to incorporate additional cultural variations, thereby improving the model performance across diverse populations.

## 6. Limitations and Future Work

While our suggested approach beat other methods overall, there is still room for development in general. Because the suggested methodology is still in development, several constraints must be addressed. Our approach, for example, is not yet resistant to all sorts of attack presentation (AP) assaults. The methodology is also computationally intensive, and it may be impractical to use on devices with limited resources. To overcome the shortcomings of the proposed methodology, in future, we intend to create more robust methods for identifying attack presentation (AP) attacks and to make the methodology more computationally efficient. We also intend to investigate the potential uses of the suggested methodology, such as detecting fake news and fraud.

## 7. Conclusions

In this research, we proposed a series of novel strategies to enhance the classification accuracy of face presentation attack detection (PAD) models, with a focus on challenging cases. Our approach expands face PAD by incorporating facial expressions and dynamic backdrops, leveraging data augmentation techniques, adversarial training, ensemble learning, fine-tuning, transfer learning, model interpretability, and attention mechanisms. Through rigorous experimentation on benchmark datasets, we demonstrated that our proposed methodology significantly outperformed existing approaches. The improvements achieved in classification accuracy can enhance the security and reliability of face recognition systems in real-world applications. Furthermore, our refined evaluation framework followed standardized preprocessing protocols, including the uniform selection of 25 frames per video, ensuring fair benchmarking with state-of-the-art PAD methods. While our method showed substantial potential, certain limitations remain. Future research should focus on addressing dataset biases, improving cross-domain adaptability, scaling to larger datasets, enhancing robustness against novel attack strategies, and enabling real-time implementation. By refining and extending the methodologies presented, we can further enhance the effectiveness and applicability of face PAD systems. Ultimately, our work contributes to strengthening the security and reliability of facial recognition systems, ensuring their robustness across diverse real-world environments.

## Figures and Tables

**Table 1:** One-class domain adaptation achievement (%).

Method	APCER	BPCER	EER
IT + OCSVM	12.4	15.2	13.8
IT + GMM	10.9	14.5	12.7
OCA-FAS	8.3	10.1	9.2
Proposed Model	6.5	8.2	7.3

**Table 2:** Client-specific domain adaptation performance (%).

Protocol	APCER	BPCER	EER
I-N-CS	6.3	8.1	7.2
C-N-CS	7.8	9.5	8.6
M-N-CS	6.0	7.8	6.9
Proposed Model (Avg.)	6.7	8.4	7.5

**Table 3:** Comparative evaluation of CIMN-OCDA protocol one-class domain adaptation techniques.

Method	HTER (%)												
	C-I	C-M	C-N	I-C	I-M	I-N	M-C	M-I	M-N	N-C	N-I	N-M	Average
IT	37.8	15.6	29.9	46.6	37.4	44.7	36.5	24.9	39.9	30.4	30.5	23.2	33.1
IT + Fine Tune	42.6	24.7	37.2	50.9	20.1	37.2	31.0	37.5	37.7	21.2	29.2	20.9	32.5
IT + OCSVM	17.7	27.4	32.9	36.3	20.5	39.3	41.2	9.9	31.9	27.7	19.6	26.9	27.6
IT + GMM	10.1	29.7	36.3	25.3	20.7	32.3	29.1	7.8	29.3	24.1	12.9	22.5	23.3
IT + OCDA	5.0	16.5	28.4	33.4	22.3	32.6	28.2	4.4	28.7	23.2	4.5	12.1	19.9
Ours	4.2	15.3	27.6	29.5	20.6	31.1	28.4	3.6	24.9	21.6	3.9	10.4	18.4

**Table 4:** Analysis of performance with one-class domain adaptation method, with FRR = 10%.

Method	HTER (%)												
	C-I	C-M	C-N	I-C	I-M	I-N	M-C	M-I	M-N	N-C	N-I	N-M	Average
IT	56.3	22.1	30.3	59.7	41.5	50.5	43.5	47.8	42.6	40.8	56.7	26.8	43.2
IT +Fine Tune	45.5	23.6	36.7	43.1	25.2	40.8	31.2	37.8	34.5	32.5	31.0	36.7	34.9
IT + OCSVM	24.5	27.5	34.8	34.9	29.4	42.3	43.3	10.7	32.1	27.2	24.7	30.3	30.1
IT + GMM	13.2	35.1	36.2	36.2	21.8	33.6	34.2	8.4	29.0	23.7	15.0	25.3	26.0
IT + OCDA	5.3	19.1	30.0	35.2	29.6	32.5	30.5	5.8	28.5	22.9	4.5	21.9	22.2
Ours	3.8	16.0	15.8	29.1	23.0	28.3	25.2	5.2	27.1	22.2	3.7	20.0	18.2

**Table 5:** Comparison analysis with unsupervised domain adaptation methods.

Method	HTER (%)												
	C-I	C-M	C-N	I-C	I-M	I-N	M-C	M-I	M-N	N-C	N-I	N-M	Average
ADDA	43.3	38.1	32.9	51.3	36.6	51.5	40.5	36.7	40.2	30.2	36.1	34.9	39.4
DRCN	45.9	29.1	34.0	50.4	43.5	51.5	30.4	38.3	40.9	33.8	38.9	38.7	39.6
DupGAN	43.7	34.9	32.3	48.0	37.7	48.5	28.6	36.9	36.0	26.1	37.4	34.9	37.1
USDAN-Un	17.5	10.7	/	31.7	27.3	/	14.8	4.9	/	/	/	/	/
KSA	40.8	16.6	33.1	13.8	36.4	41.6	10.6	34.8	31.9	31.6	40.3	27.6	29.9
ADA	19.0	10.8	30.9	43.0	32.0	43.2	19.2	6.6	34.2	35.6	31.8	33.0	28.3
ML-Net	44.8	15.5	33.9	46.9	36.8	44.3	39.3	13.0	36.1	27.2	32.2	34.1	33.7
UDA	17.1	10.5	29.5	35.7	30.5	41.3	18.3	4.5	31.2	19.4	25.2	25.9	24.1
CIMN	5.0	16.5	28.4	33.4	22.3	32.6	28.2	4.4	28.7	23.2	4.5	12.1	19.9
Ours	3.8	12.0	25.1	20.2	18.9	33.4	22.8	3.6	26.1	20.6	3.9	9.8	16.6

**Table 6:** Performance comparison with conventional methods.

Methods	ACER (%)	AUC (%)
DTN	15.61 ± 1.67	/
OCA-FAS	2.26 ± 0.39	/
IT	3.95 ± 0.30	98.17 ± 0.17
OCDA	0.46 ± 0.12	99.63 ± 0.12
Ours	0.28 ± 0.07	99.89 ± 0.07

**Table 7:** Analysis using HTER matrix.

PROTOCOLS	Method	HTER (%)										
		Client-1	Client-2	Client-3	Client-4	Client-5	Client-6	Client-7	Client-8	Client-9	Client-10	Overall
I-N-CS	IT	38.16	49.42	36.72	35.71	29.49	48.00	49.95	46.18	42.60	47.51	42.87 ± 8.07
	IT + Fine Tune	25.99	24.52	225.57	19.97	27.35	16.15	36.25	24.42	35.42	27.50	28.61 ± 6.43
	IT + OCSVM	26.55	21.83	15.53	9.19	16.20	20.07	24.41	13.80	16.11	29.49	19.32 ± 6.29
	IT + GMM	24.13	20.38	18.72	10.96	17.56	15.87	26.45	20.71	16.12	25.37	19.63 ± 4.81
	IT + OCDA	18.97	18.20	13.05	7.89	16.21	11.53	20.13	14.13	10.22	16.24	14.66 ± 4.00
	Ours	14.78	13.99	11.67	4.24	10.63	9.41	18.69	12.32	8.73	13.91	11.84 ± 3.10
M-N-CS	IT	46.13	39.57	36.49	45.14	39.05	42.49	31.79	34.93	42.25	35.93	39.28 ± 4.60
	IT + Fine Tune	24.32	21.09	16.54	13.04	27.42	27.55	16.49	15.55	13.97	21.64	20.76 ± 5.39
	IT + OCSVM	21.61	18.47	11.68	13.79	13.38	24.56	17.17	13.66	11.41	19.81	16.55 ± 4.48
	IT + GMM	21.50	17.63	11.74	11.64	15.57	19.61	21.26	14.99	11.53	19.92	16.55 ± 4.02
	IT + OCDA	12.34	18.06	7.92	8.13	13.41	14.23	13.84	11.01	10.98	16.54	12.65 ± 3.29
	Ours	10.76	16.56	6.89	7.16	11.97	12.39	9.64	8.32	11.77	14.54	11.0 ± 2.01
C-N-CS	IT	33.39	29.11	23.68	33.88	26.54	28.48	30.60	32.35	29.14	29.53	30.27 ± 3.14
	IT + Fine Tune	24.83	25.48	18.19	14.27	26.59	19.73	13.86	17.37	20.39	18.50	19.12 ± 4.89
	IT + OCSVM	23.17	23.03	11.39	7.40	17.67	21.87	13.25	17.80	12.48	25.19	17.33 ± 5.99
	IT + GMM	21.04	20.12	7.16	6.51	20.24	16.20	22.74	19.00	13.82	21.71	16.85 ± 5.90
	IT + OCDA	6.22	9.89	3.75	6.07	8.05	10.19	14.94	6.43	10.96	9.14	8.56 ± 3.18
	Ours	4.62	6.94	2.07	5.01	6.91	7.79	11.76	3.61	6.01	3.10	5.78 ± 2.74

**Table 8:** Performance comparison using a HTER Matrix with one-class domain adaptation with CIM-N-CS-OSDA protocol.

Protocols	Method	AUC (%)										
		Client-1	Client-2	Client-3	Client-4	Client-5	Client-6	Client-7	Client-8	Client-9	Client-10	Overall
I-N-CS	IT	66.56	47.10	74.95	73.66	82.55	45.58	49.95	59.65	59.00	56.14	58.21 ± 12.48
	IT + Fine Tune	79.98	81.02	82.25	90.19	79.43	93.66	66.37	82.05	69.36	81.16	80.55 ± 8.15
	IT + OCSVM	82.05	85.51	93.19	95.56	90.15	85.59	79.18	91.26	89.58	77.24	86.93 ± 6.07
	IT + GMM	81.30	85.98	89.72	94.29	87.81	88.30	79.97	86.70	88.22	81.94	86.42 ± 4.34
	IT + OCDA	89.54	88.92	92.61	96.92	89.05	92.62	86.53	93.29	95.61	92.16	91.73 ± 3.22
	Ours	94.89	92.78	96.39	98.21	92.13	94.01	89.78	96.15	98.03	96.54	93.89 ± 1.99
M-N-CS	IT	58.01	63.94	62.60	60.13	62.98	60.40	77.97	73.60	63.29	68.07	65.10 ± 6.32
	IT + Fine Tune	81.99	84.08	90.84	93.37	79.01	74.73	89.66	93.83	92.48	84.16	86.42 ± 6.60
	IT + OCSVM	85.80	88.63	94.51	93.09	93.93	82.72	90.67	91.02	94.66	85.18	90.02 ± 4.27
	IT + GMM	85.90	89.66	94.91	95.42	90.71	88.22	83.70	92.56	93.29	88.65	90.30 ± 3.83
	IT + OCDA	93.83	90.64	97.33	97.70	89.98	93.29	90.32	95.62	95.79	91.74	93.62 ± 2.90
	Ours	95.69	91.98	98.34	99.50	91.09	95.89	93.68	97.14	96.89	93.64	95.98 ± 1.56
C-N-CS	IT	74.22	77.39	86.52	73.49	77.40	78.65	80.52	76.77	78.66	80.63	78.43 ± 3.68
	IT + Fine Tune	82.06	85.69	88.93	91.22	84.28	85.56	89.87	90.25	82.76	88.50	86.91 ± 3.26
	IT + OCSVM	82.89	83.81	95.73	96.16	90.13	86.38	91.41	87.03	91.53	80.66	88.57 ±5.30
	IT + GMM	85.65	84.87	96.57	96.84	88.41	91.97	80.72	86.35	91.22	86.37	88.90 ± 5.20
	IT + OCDA	97.52	95.31	99.18	98.44	96.20	96.12	91.66	98.42	96.72	96.70	96.63 ± 2.13
	Ours	98.98	98.13	99.91	99.58	97.01	98.78	94.83	99.79	98.67	99.65	98.69 ± 1.89

## Data Availability

The data used in this study is publicly available from the following sources: Replay-Attack Database: A dataset containing 1300 video clips of photo and video attack attempts on 50 clients under different lighting conditions. Available at: https://www.idiap.ch/en/scientific-research/data/replayattack (accessed on 16 March 2025). NTU 384 (ROSE-Youtu): A face liveness detection dataset provided by the ROSE Lab at Nanyang Technological University. Available at: https://rose1.ntu.edu.sg/dataset/faceLivenessDetection/ (accessed on 16 March 2025). MSU-MFSD: A dataset for mobile face spoofing detection. Available at: https://github.com/sunny3/MSU-MFSD?tab=readme-ov-file (accessed on 16 March 2025). OULU-NPU Database: A mobile face presentation attack database with real-world variations. Available at: https://sites.google.com/site/oulunpudatabase/ (accessed on 16 March 2025). CASIA-FASD: A face anti-spoofing dataset available on Kaggle. Available at: https://www.kaggle.com/datasets/immada/casia-fasd (accessed on 16 March 2025). These datasets were used for training and evaluation of the proposed model.
